# Cancer susceptibility and reproductive trade-offs: a model of the evolution of cancer defences

**DOI:** 10.1098/rstb.2014.0220

**Published:** 2015-07-19

**Authors:** Amy M. Boddy, Hanna Kokko, Felix Breden, Gerald S. Wilkinson, C. Athena Aktipis

**Affiliations:** 1 Department of Psychology, Arizona State University, Tempe, AZ, USA; 2 Center for Evolution and Cancer, University of California San Francisco, San Francisco, CA, USA; 3 Wissenschaftskolleg zu Berlin, Institute for Advanced Study, 14193 Berlin, Germany; 4 Institute of Evolutionary Biology and Environmental Studies, University of Zurich, Winterthurerstrasse 190, 8057 Zurich, Switzerland; 5 Department of Biological Sciences, Simon Fraser University, Burnaby, British Columbia, Canada V5A 1S6; 6 Department of Biology, University of Maryland, College Park, MD 20742, USA

**Keywords:** cancer defences, life-history trade-offs, reproductive competition, sexual selection, comparative oncology

## Abstract

The factors influencing cancer susceptibility and why it varies across species are major open questions in the field of cancer biology. One underexplored source of variation in cancer susceptibility may arise from trade-offs between reproductive competitiveness (e.g. sexually selected traits, earlier reproduction and higher fertility) and cancer defence. We build a model that contrasts the probabilistic onset of cancer with other, extrinsic causes of mortality and use it to predict that intense reproductive competition will lower cancer defences and increase cancer incidence. We explore the trade-off between cancer defences and intraspecific competition across different extrinsic mortality conditions and different levels of trade-off intensity, and find the largest effect of competition on cancer in species where low extrinsic mortality combines with strong trade-offs. In such species, selection to delay cancer and selection to outcompete conspecifics are both strong, and the latter conflicts with the former. We discuss evidence for the assumed trade-off between reproductive competitiveness and cancer susceptibility. Sexually selected traits such as ornaments or large body size require high levels of cell proliferation and appear to be associated with greater cancer susceptibility. Similar associations exist for female traits such as continuous egg-laying in domestic hens and earlier reproductive maturity. Trade-offs between reproduction and cancer defences may be instantiated by a variety of mechanisms, including higher levels of growth factors and hormones, less efficient cell-cycle control and less DNA repair, or simply a larger number of cell divisions (relevant when reproductive success requires large body size or rapid reproductive cycles). These mechanisms can affect intra- and interspecific variation in cancer susceptibility arising from rapid cell proliferation during reproductive maturation, intrasexual competition and reproduction.

## 1. Introduction

Cancer incidence across species varies widely. These differences in cancer susceptibility are due to both environmental exposures [1], and evolutionary pressures shaping organism-level cancer susceptibility and cancer defences [2–4]. Beneficial traits such as defence against disease are often costly, and such trade-offs can maintain genes that contribute to disease susceptibility [5]. For example, trade-offs between reproduction and immune defence have been observed in a number of species [6,7]. Similar trade-offs have been proposed to underlie observed associations between reproductive competitiveness and cancer [8], though as of yet there has been no formal model describing these trade-offs and the factors affecting them.

Accumulating genetic and molecular evidence suggests that trade-offs between benefits early in life and disease susceptibility later in life may be widespread across disease (as reviewed in Carter & Nguyen [9] and Leroi *et al*. [10]). This phenomenon may also significantly shape the susceptibility to cancer [9]. Cancer suppressor genes such as P53 [11,12] and BRCA [13] play important roles in fertility and may be maintained owing to trade-offs between fertility enhancement and cancer risk.

Trade-offs between reproductive competitiveness and cancer risk can be expected for two main reasons: (i) selection for mechanisms that enable rapid cell proliferation could simultaneously enhance extreme trait expression as well as tumour formation and (ii) selection for increased allocation of energy to reproductive traits rather than to somatic maintenance could elevate cancer risk by increasing somatic mutation rates. The first type of trade-off may involve mutations or epigenetic silencing of cancer suppression genes. The second type of trade-off could be due to altered allocation of a finite energy pool towards reproduction at the expense of somatic maintenance, such as DNA repair or immune defences. Consequently, mutations that increase cancer risk arise more easily if energy must be diverted away from DNA repair to support development or expression of reproductive traits. These two pathways are distinct, but can potentially interact to produce trade-offs between reproductive competitiveness and cancer susceptibility. Even if somatic mutation rate per cell division did not change, reproductive success might require a larger number of cell divisions (e.g. when sexual selection favours large-bodied males), and this again increases the total risk.

In this paper, we explore the effect of reproductive trade-offs on the evolution of cancer defences, modelling reproductive competitiveness as the extent to which the most competitive individuals dominate reproduction. Reproductive competitiveness is an important force in the evolution of extreme morphologies and life histories, and is often impacted by sexual selection [14]. Traits such as large body size, extreme morphology (i.e. weapons or ornaments), larger and more frequent litter size, and aggressiveness can all have positive effects on fitness through preferential mating or differential fitness owing to higher reproductive output, but they can also be costly in terms of increasing mortality or morbidity. Large body size and extreme morphologies often require greater levels of cell proliferation via an increase in growth signalling mechanisms (including hormones and growth factors). Additionally, factors that accelerate organismal reproduction (e.g. growing fast, early maturation) or increase the frequency of reproduction might enhance cancer risk through the allocation of energy towards reproduction rather than through somatic maintenance, potentially increasing the likelihood of mutations in cancer suppressor genes.

Our model does not explicitly distinguish between these two types of trade-offs (cell-proliferation-related vulnerabilities and energy allocation trade-offs). For simplicity and generality, we base our model on the intensity of the trade-off between reproductive competitiveness and cancer defences. In addition to varying the intensity of the trade-offs, we also consider varying levels of extrinsic mortality. This allows us to explore the evolutionary viability of cancer defences in species under different reproductive, social and ecological conditions. Mortality (and the consequent expected lifespan) is important, because cancer defences can delay the onset of cancer, but delays bring about little selective benefit at an age where the organism is likely to have died of other causes. We use this model to make predictions about the patterns of cancer incidence across species and review a variety of mechanisms that might underlie trade-offs between reproductive competitiveness and cancer defence.

## 2. Model

### (a) Stronger intraspecific competition is predicted to lead to higher cancer incidence

Can reproductive competition within a population lead to individuals adaptively ‘neglecting’, or never evolving, some of the possible defences against cancer? Here, we derive an optimality model to address this question, as well as whether populations with particularly strong intraspecific competitive effort should be expected to have an elevated risk of mortality owing to cancer (as opposed to some other cause). In this model, the population can refer to all individuals, regardless of their sex, but it is also possible to make statements about a subset of the biological population to the extent that competition occurs among ‘peers'. For example, the population may be split into males and females, which means that other males form the relevant peer group of males, whereas females compete with other females. Consequently, we also consider whether male defences against cancer are expected to differ from those of females.

### (b) Model assumptions and derivations

#### (i) Cancer defence

First, we assume that variation in the strength of defences against cancer is present in the population. The model does not specify how the defence acts to delay cancer onset. Instead, we use an operational definition. Assuming that cancer takes *n* steps to form, with each occurring at a rate *k_i_* per cell, where *i* = 1, 2, … , *n*, if *n* steps have occurred in a particular cell lineage, then the whole organism has cancer. The organism possesses defence levels *d_i_* against each step *i*, where 0 ≤ *d_i_* ≤ 1. Each *d_i_* denotes the reduction in the rate at which the corresponding step occurs: if, for example, *d_i_* = 0.5, the *i*th step takes, on average, twice as long to happen than it would in the absence of any defence; if *d_i_* = 0.75, it takes four times as long (only 25% of the rate then remains, and 1/0.25 = 4). One possible way to achieve *d_i_* = 0.5 is to have DNA repair remove half of the critical mutations, but less direct ways include efficient immunocompetence that clears half of the infections before they can heighten the organism's cancer risk. Alternatively, the organism may simply eliminate half of the precancerous cells.

For simplicity, our examples are built assuming that all limiting steps occur at the same rate *k_i_* = *k* for all *i*, and that cancer defence likewise is the same across steps, *d_i_* = *d* for all *i*. Without this assumption, the analysis would proceed as below but with specific multiplications for each *i* separately. The general model intends to make no statements about the relative efficiency of particular defence types against specific rate-limiting steps.

#### (ii) Reproductive competition within the peer group

Next, we specify how intraspecific competition impacts an individual's reproductive success. As described in §1, trade-offs between defence and investment in immediate reproductive success may occur for multiple reasons. For example, the latter may require fast growth, but this comes at a cost to the former. We assume competitiveness, *c*, is a function of two parameters *α* (the intensity of intraspecific competition) and *β* (the strength of trade-offs): the rate of fitness accumulation per time unit spent alive and cancer free is assumed proportional to *c^*α*^* = (1 – *d^*β*^*)*^*α*^*.

This is specified as ‘proportional to’ rather than as ‘equal to’ to take into account the fact that fitness is relative to conspecifics. Thus, if surrounded by many other competitive individuals, fitness of the focal individual may remain low, but for the subsequent analysis contrasting alternative life histories of a focal individual the effect of others stays constant and does not have to be included [15]. Here, *c* = 1 – *d^*β*^* describes the individual's competitiveness assuming it does not yet have cancer. Cancerous individuals are assumed to be non-competitive and thus gain zero reproductive success while alive with cancer.

Competitiveness, as defined above, is a declining function of cancer defences, which reflects the assumption that defences are costly. The parameter *α* describes the intensity of intraspecific competition (in many cases, limited to competition within a sex, i.e. intrasexual competition) such that resources obtained scale with competitiveness according to a power function c*^*α*^* (see parameter *u* in an analogous treatment in [15]). Thus, if *α* = 1, an individual whose competitiveness *c* exceeds that of its peer group by a certain percentage (say, 10%) will have an equivalently better (in this example 10%) access to resources. If *α* < 1, the benefits of increased competitiveness are lower: a 10% increase in *c* would, then, lead to lower than 10% improvement in reproductive gains. Conversely, *α* > 1 will make the resource–competitiveness relationship steeper, so that if *α* ≫ 1, competition is of a ‘winner-takes-all’ nature: a higher competitiveness, *c*, compared with the rest of the peer group will then lead to the focal individual reaping almost all the reproductive benefits.

The parameter *β* changes the shape of trade-offs: if *β* is small, then competitiveness declines in response to investing in other life-history components (here, cancer defence) even if the defence is not intensive; if *β* is large, then individuals can retain their competitiveness intact up to high (*d* ≈ 1) defence levels.

Individuals are assumed to gain reproductive payoffs (of magnitude *c^*α*^*) at a constant rate as long as they are cancer-free and have also avoided other sources of mortality.

#### (iii) Cancer-free lifespan

There are two ways in which an individual can end its reproductive career. The first is death owing to a source of mortality other than cancer, which we assume occurs at a constant rate, *μ. A*(*t*) is the probability that this type of death has happened by time *t*. The second way an individual can die is by cancer, that is, when one of the *N* cell lineages has passed through all the rate-limiting steps, denoted by *B*(*t*), the probability that this has happened by time *t*. The overall probability that the individual is alive is2.1
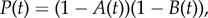
where2.2
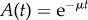
and2.3
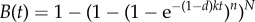
*B*(*t*) is formed in the following way: 1 − e^–(1 −^ *^d^*^)*kt*^ is the probability that a single rate-limiting step has already happened by time *t*. When we assume that *n* steps are required before oncogenesis can end an individual's reproductively active life, (1 − e^−(1−*d*)*kt*^)^*n*^ is the probability that all of them have happened in the same focal cell lineage. The complementary probability 1 − (1 − e^−(1−*d*)*kt*^)^*n*^ consequently gives the probability that the focal lineage is healthy at time *t*. If there are *N* such lineages in an organism, (1 − (1 − e^−(1−*d*)*kt*^)^*n*^)^*N*^ (which raises the above to the power *N*) is the probability that all cell lineages are healthy at time *t.* The complement of this quantity is the probability *B*(*t*) of cancer having already emerged in at least one cell lineage as indicated above.

**Figure 1.**
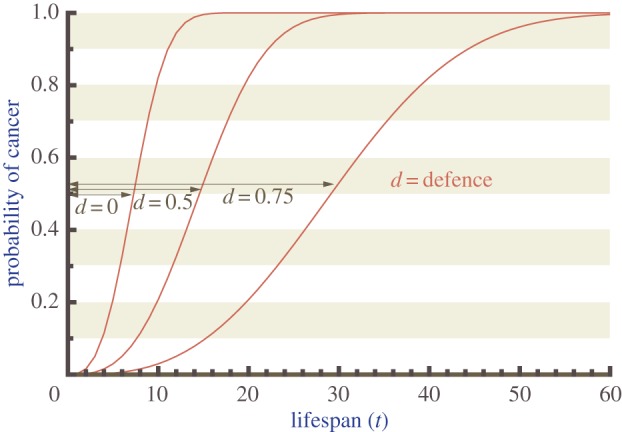
Higher cancer defence increases lifespan. The probability that cancer has arisen by age *t* for three different defences *d* = 0, *d* = 0.5 and *d* = 0.75. Note that for any probability level, *d* indicates the proportion of the arrow that extends beyond the *d* = 0 curve. Thus, e.g. with *d* = 0.75, three quarters of the delay from birth to cancer is due to the defence effort, one quarter owing to the time that mutations take to occur even if there is no cancer defence effort. Graphs are derived using parameters *k* = 0.01, *n* = 3 and *N* = 2000.

In the absence of any defences against cancer, the probability that the individual has cancer by time *t* rises from zero to unity as time passes (figure 1). The effect of defence is to delay this increase in a very specific manner: for each value of *d*, the cumulative probability curves of cancer having ended the individual's cancer-free lifespan are shifted to the right such that any horizontal time line drawn from *t* = 0 to the probability curve has exactly the proportion *d* spent in the state of ‘cancer-free life prolonged due to defences'. Thus, if, for example, *d* = 0.75, one-quarter of the age that the organism managed to spend cancer-free was caused by ‘luck’, whereas three-quarters can be attributed to defences delaying the eventual inevitability of cancer.

To sum up the effect of *d* on realized lifespan, however, it is not sufficient to stop at the information contained in figure 1, because other sources of mortality can make it unlikely that the organism is alive to reap the probabilistic benefits of the longest delays at the upper end of figure 1. In this context, it is insightful to use equation (2.3) to derive the time, denoted *t*(*P, d*), that it takes cancer incidence to reach any pre-specified value *P*, for a given level of defences *d*2.4
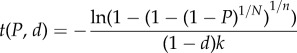


**Figure 2.**
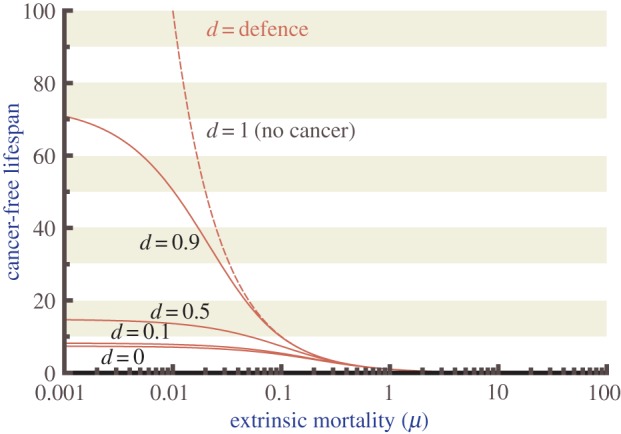
The effects of cancer defences on lifespan are strongest when extrinsic mortality is low. Model predictions for the expected duration of cancer-free life, which can be ended by either cancer or through other sources of mortality, as a function of extrinsic mortality *μ*, plotted for different values for defence effort *d* as indicated on the graph (lowest curve: *d* = 0, then *d* = 0.1, followed by *d* = 0.5 and *d* = 0.9). The dotted line gives the expected lifespan assuming no cancer ever occurs (*d* = 1). The impact of defence on lifespan varies from strong when extrinsic mortality is low, to negligible when lifespans are short owing to causes other than cancer. Parameters as in figure 1. Note that neither *α* nor *β* impact the graphs in figure 1 or in this figure.

Expected lifespan then is an integral2.5
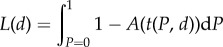
Numerical integration of *L*(*d*) leads to a crucial insight. If extrinsic mortality (*μ*) is high (right side of figure 2), investing in defence, *d*, does not prolong lifespan. Even in the absence of defences, cancer remains an insignificant cause of death as it does not ‘have the time’ to develop before the organism has already been killed by predators or any other factors categorized as extrinsic mortality (*μ*). Only when these other sources of mortality are low (left side of figure 2), does investing in defence, *d*, have a substantial impact on lifespan. This highlights that in our aim to derive how optimal cancer defences depend on intraspecific competition (parameters *α* and *β*), we also must consider scenarios with a variety of extrinsic mortalities (*μ*).

**Figure 3.**
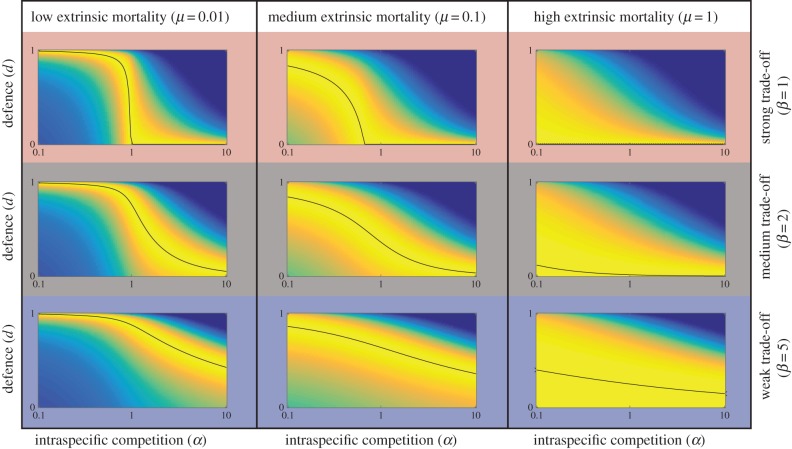
Intraspecific competitiveness has the strongest effect on cancer risk in environments with low extrinsic mortality and strong trade-offs. When extrinsic mortality is low (*μ* = 0.01) and trade-offs between cancer defence and reproductive competition is high (*β* = 1), as intraspecific competition increases (*α*), cancer defences remain high until a critical threshold is reached (when intraspecific competition is greater than 1) and then dramatically declines (upper left). Cancer defence declines in all cases with *α*, but is sometimes low to begin with (upper right corner where extrinsic mortality is high). Parameters as in figure 1 and figure 2, and additionally *β* = 1, 2 or 5 (top, middle and bottom row, respectively), and *μ* = 0.01, 0.1 or 1 (left, middle and right column, respectively). Colours indicate the fitness consequences of each possible level of defence effort (0 ≤ *d* ≤ 1 as indicated on the *y* axis) for value of intraspecific competition (*α*) ranging from 0.01 to 10. More yellow indicates higher fitness, more blue low fitness; the black line additionally indicates highest fitness (optimum).

**Figure 4.**
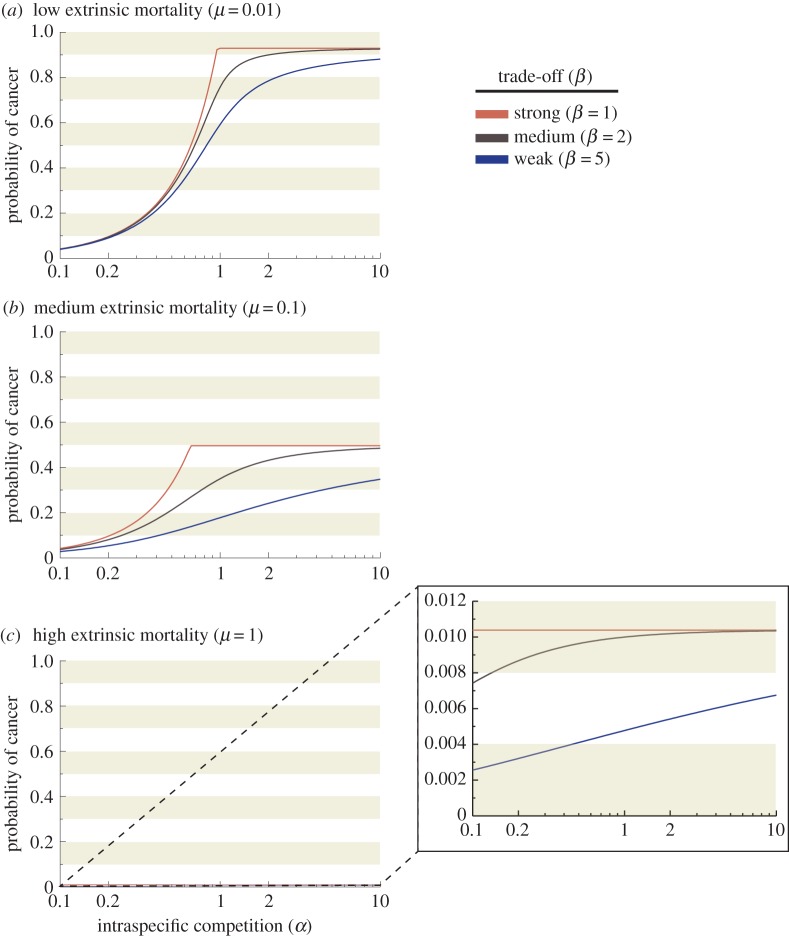
Stronger intraspecific competition increases the probability of cancer. The incidence of cancer, measured as the probability of cancer rather than extrinsic mortality ending an individual's reproductively active life, increases as a function of reproductive competition (*α*) under three different extrinsic mortalities (*a*) *μ* = 0.01, (*b*) *μ* = 0.1, (*c*) *μ* = 1, and three values of *β* (uppermost curve within each figure: *β* = 1, middle curve: *β* = 2, lowest curve: *β* = 5). Incidence always increases with intraspecific competition (*α*), but in (*c*) where extrinsic mortality is high, the increase does not yield high cancer incidence for any value of intraspecific competition (*α*).

To derive how optimal cancer defences depend on intraspecific competition, while considering scenarios with a variety of extrinsic mortalities (*μ*), we compute lifetime fitness, i.e. the quantity (1 – *d^*β*^*)^α^*L*(*d*), for different extrinsic mortalities and intraspecific competition (*α*) and trade-off (*β*) values and find the maximum. The results (figure 3) show that in all the mortality scenarios (*μ* = 0.01, 0.1 and 1) and whether we assume weak or strong trade-offs (*β*) between cancer defences and reproductive success, the optimal defence always declines with increasing intraspecific competition (*α*), unless defence is already low to begin with (top right figure). The results remain similar across all values of *n* and *N* that we tested, though for simplicity only one set of parameters is shown. Lastly, we present alternative extrinsic mortality (*μ*) scenarios and illustrate how often life is actually ended by cancer, as opposed to all other causes. More intense intraspecific competition (*α*) increases mortality risk by cancer unless trade-offs (*β*) are low (figure 4).

## 3. Predictions from the model

### (a) High intraspecific competition lowers cancer defences

Our model predicts that individuals may evolve traits that increase reproductive success even if this increases their cancer risk, but the extent to which this applies depends on the intensity of intraspecific (often intrasexual) competition as a determinant of reproductive success, and also other parameter values. In an environment with low extrinsic mortality (*μ* = 0.01), we expect more cancer in species where intraspecific competition (*α*) is very strong (at the extreme, of a winner-takes-all nature; figure 3 top left). Species in high extrinsic mortality environments (*μ* = 1), on the other hand, are expected to die from other causes than cancer, regardless of the intensity of intraspecific competition (*α*) (figure 4*c*). If extrinsic mortality is lower, then cancer incidence can depend strongly on the role that intraspecific competition (*α*) plays in shaping life histories (figure 4*a,b*). Intraspecific competition can vary between males and females, and thus we will discuss our predictions and review of the literature separately for each sex.

#### (i) Sexually selected traits may increase cancer risk in highly competitive males

**Figure 5.**
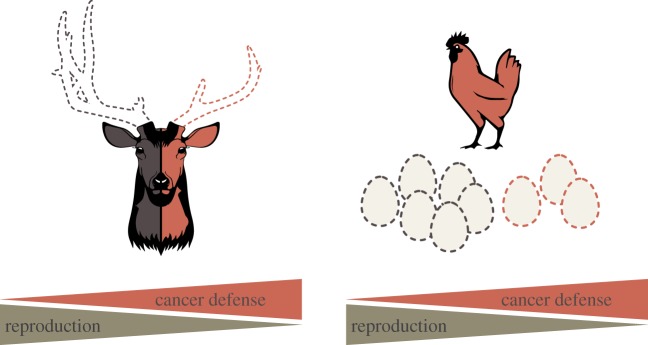
Increased reproductive effort through mating effort or fertility may lower cancer defences. An illustration of our hypothesis–selection to enhance reproductive competition decreases cancer defences. Traits selected to increase reproductive effort include body size, ornaments or weapons in males, or fertility frequency and productivity in females. Hypothetical trade-offs are illustrated above depicting examples of reproductive competition. For example, the smaller antler on the deer illustrates less reproductive competitiveness, but better protection against cancer. Similarly, increased reproductive output in domesticated chickens may trade off with lower cancer defences.

Males often invest more energy in obtaining a mate than females and this can lead to high competition among individuals to reproduce. For example, males with high reproductive competitiveness participate in male–male competition and dominant males gain more opportunities to mate. This competition can favour the evolution of secondary sexual characteristics (e.g. extreme traits) [14,16], and it is well known that a shorter lifespan may be an ‘acceptable’ cost of such traits in the sense that net selection favours their exaggeration, whereas male lifespan is shortened. According to our model, we predict sexually dimorphic species, such as individuals with extreme ornaments and weapons or larger body size, to be subject to increased cancer risk (figure 5); similarly, we expect more cancer in species with strong sperm competition (particularly in the relevant tissues such as the testes). These trade-offs may be a result of vulnerabilities arising from increased cell proliferation (e.g. growth promoting signals to produce large body size or weapons; more of the continually dividing cells in larger testes) or as a result of allocation of energy towards reproductive competition at the expense of somatic maintenance (e.g. DNA repair, immune defences). However, it is clearly difficult to obtain cancer incidence for species in the wild, especially when male–male competition is intense: poor performance of an individual after the onset of cancer can lead to a death (e.g. taken by a predator) that is not easy to trace back to cancer.

There is some evidence to suggest that selection for large body size in males can lead to higher cancer susceptibility. For example, *Xiphophorus maculatus*, a freshwater fish called the Southern platyfish, carries a dominant oncogene (Xiphophorus melanoma receptor kinase, *Xmrk*) that is normally suppressed but can result in the formation of male-biased malignant melanomas in hybrids [17–20]. Body size can strongly predict reproductive success in this species, and the *Xmrk* genotype is positively correlated with a larger body size in both males and females. Females prefer to mate with males carrying a larger black spot [17,18], and males with melanoma and *Xmrk* were significantly longer than both males with *Xmrk* and no melanoma and males without *Xmrk* [17]. *Xmrk* is derived from a gene duplication event of an epidermal growth factor receptor that controls cell proliferation [21], and it may be involved in binding to growth factor ligands that increase both body size and melanoma formation in several *Xiphophorus* species [22].

Another intriguing example of a trait that can enhance reproductive competitiveness of males, but also may increase cancer risk is the production of sexually selected weapons, such as antlers. In most deer species, only males grow antlers, and growth is initiated at puberty [23,24]. New antler growth and casting is seasonal, and thought to be controlled by levels of circulating testosterone [25,26]. It has been hypothesized that there is also a period of androgen-independent growth during antler formation [23], where circulating levels of insulin-like growth factor-1 (IGF-1) were significantly increased in male deer growing their antlers [27]. Male–male competition can lead to strong selection for growth to produce such elaborate traits, but this comes with a risk: disregulation in the system can lead to uncontrolled cellular growth. Species with antlers are accordingly susceptible to antleromas, which are massive growths found on the antlers of free ranging deer [28–35]. Antleromas can be artificially produced when androgen production is disrupted (e.g. castration) and circulating testosterone does not cycle [32], suggesting that interactions between hormone levels and growth factors underlie the mechanisms creating susceptibility to antleromas.

#### (ii) Factors that enhance female fertility may lead to elevated cancer risk

In many species, females invest typically more heavily in other components of reproductive effort rather than mating effort. Determinants of reproductive success for females often include components such as fertility timing and frequency, as well as size of the litter and investment in individual offspring. Our model is not sex-specific *per se*: although intraspecific competition (*α*) is probably lower for most females compared with males (reproduction is not of a ‘winner-takes-all’ nature), it still predicts that investment in traits that elevate reproductive competitiveness can elevate susceptibility to cancer, especially if very high reproductive output requires rapid cell proliferation (figure 4). Given that early reproductive maturity and rapid offspring production both require rapid cell proliferation, and may also involve the allocation of energy towards reproduction at the expense of DNA repair, we consider it possible that selection for improved reproductive success can lead to higher cancer risk in females, too.

Once again, detecting cases in the wild remains challenging: natural selection can yield life histories where most deaths occur before the expected onset of cancer (figure 4). Artificial breeding, however, offers instructive examples for revealing underlying trade-offs: intense selection for reproductive traits can heighten cancer risk considerably (figure 5). The domesticated Jungle fowl hen (*Gallus gallus domesticus*) has been artificially selected for daily ovulation and continuous egg-laying. This hen is the only known non-human animal with a high incidence of spontaneous ovarian cancer [36]. Ovarian cancer is observed in hens as young as 2 years of age, and 30–35% develop the highly malignant disease by 3.5 years of age [36,37].

## 4. Discussion

### (a) Life-history trade-offs between cancer defences and reproduction

One way reproductive competition may be lowering cancer defences is through energy allocation trade-offs [38]. Organisms have limited energy to divide between soma maintenance and reproduction, with allocations dependent on life history (see ‘disposable soma theory’ [39]). Short-lived organisms allocate more resources towards reproduction in order to reproduce successfully within their lifespan, whereas investment in somatic maintenance (among them cancer defences) has allowed species to extend their lifespans [40,41]. When all else is equal, short-lived rodents are more prone to cancer [42,43] than are long-lived species, such as elephants and whales [3]. Indicative of lower defences, cells of shorter-lived organisms overcome intrinsic growth limitations more easily (i.e. transformation in cell culture) [44], allocate less time to DNA repair (short-lived species have more unresolved DNA damage in the form of micronuclei) [45] and proliferate rapidly *in vitro* [46]. Our results suggest that large-bodied animals *must* invest more in somatic maintenance, including tumour suppression, to build and maintain a large body for a sufficiently long time for reproduction to occur [3,47]. It has also been demonstrated that DNA break recognition is better in mammals with long lifespans [48]. Increasing lifespan may thus select for new tumour suppressor mechanisms or a shift in energy allocation towards more prolonged somatic tissue maintenance [49].

Some of the genetic and molecular mechanisms that mediate trade-offs between cancer defences and reproduction are becoming better understood. For example, the cancer suppressor genes *P53* [11,12] and *BRCA* [13] are likely candidates given that mutations in these genes appear to be associated with enhanced fertility in some cases. Current evidence suggests that the *P53* family of tumour suppressors (which includes also p63 and p73) may be involved in fertility and reproduction as well as cell cycle regulation [50]. Mouse models have demonstrated that p63 may have effects on the quality and survival of the oocyte pool [51], p73 plays a role in early blastocyst division [52], and p53 may help regulate the implantation of the fertilized egg as well as litter size in knockout mice [53].

DNA repair is a critical mechanism for cancer suppression and somatic maintenance [54,55]. *BRCA1* and *BRCA2* (breast cancer susceptibility genes 1 and 2) genes encode for tumour suppressor proteins important in DNA repair pathways [56]. Mutations in these genes increase the risk for breast and ovarian cancers [57]. Interestingly, one study demonstrated that women with *BRCA1* and *BRCA2* mutations have greater susceptibility to breast cancer, but also higher fertility. Women with *BRCA* mutations had significantly more children, shorter birth intervals and end childbearing later than the controls [13]. However, the mechanism for increased fertility among women with *BRCA* mutations is unknown and murine models of *BRCA* mutations do not support these findings [58].

Another potential example of an association between fertility and cancer risk involves the Kisspeptin (*KISS1*) gene in humans. Kisspeptin is a G protein coupled receptor ligand that is required for normal maturation and puberty. Defects in *KISS1* lead to the absence of sexual maturation, indicating a critical role in the timing of puberty [59,60]. Kisspeptin also induces production of luteinizing hormone (LH) and follicle-stimulating hormone (FSH) and is required for menstruation. Interestingly, *KISS1* was first discovered as a metastasis suppressor gene and thought to play a role in suppression of metastasis of breast cancers and melanomas [61]. Recently, *KISS1* has been also implicated in the inhibition of trophoblast invasion and angiogenesis in the placenta [62,63]. Although the exact mechanisms are yet to be determined, it may be that the capacity to suppress trophoblast invasion might also confer capacities to suppress cell invasion more generally and thus reduce the risk of invasive/metastatic cancer.

Similarities in the underlying biological mechanisms of cancer invasion and deep placentation, such as degradation of the extracellular matrix and angiogenesis, suggest an evolutionary link between these processes [64]. Effective placental invasion may increase fertility at the cost of higher cancer risk. Given that eutherian mammals vary in the degree of placental invasiveness, cancer risk would be expected to covary, and some evidence supports this prediction. Equines and bovines have the least invasive placenta type (epitheliochorial placentation) and were shown to have lower rates of metastatic cancer than felines and canines, who have characteristics of deeper placentation (endotheliochorial placentation) [65]. Shallower placentation has evolved numerous times across eutherian mammals [66], suggesting that females of those species have also evolved mechanisms to suppress trophoblast invasion, and these may be the same underlying mechanisms that suppress metastatic disease. Additional studies, linking metastatic cancer risk and placentation penetration using species with all placental types, including those with the most invasive haemochorial placentas, are needed to confirm this relationship.

### (b) Environmental and ecological factors affect the strength of trade-offs

The strength of a trade-off between cancer defence and reproduction may be dramatically affected by ecological conditions, especially available resources (when the trade-off is due to energy allocation). Low resource levels have been found to increase the intensity of trade-offs between reproduction and immune function in a number of species [6], including the immunosuppressive effect of testosterone [67]. Differences in the intensity of trade-offs between reproduction and survival have been found in human populations with varying levels of socio-economic status, with more intense trade-offs between reproduction and longevity occurring in lower socio-economic status groups [68–70]. This suggests that environmental and ecological conditions are likely to play important roles in modulating the intensity of trade-offs between reproduction and cancer defence, especially when this trade-off is mediated by energy allocations.

In our model, we explored the implications of varying the intensity of the trade-off (*β*; figure 3). We found that in conditions with higher trade-offs owing to decreased resource availability (upper panels), elevated levels of cancer defence result in very low fitness, especially when extrinsic mortality is high. In contrast, in conditions with less intense trade-offs, corresponding to more plentiful resources, high levels of cancer defence result in extended lifespan and higher fitness. The importance of both resource levels and extrinsic mortality (which for non-humans may be caused by predation or other ecological factors) in determining the fitness of varying levels of cancer defence suggests that future work should consider these trade-offs in their ecological context.

### (c) Selection for rapid growth may mediate sexually selected traits and cancer risk

Rapid growth may not only enhance within-species reproductive competitiveness, but also lead to greater cancer risk because of vulnerabilities associated with rapid cell proliferation. The development and expression of many key aspects of morphology and physiology influenced by sexual selection and reproduction are mediated by growth factors, including steroid hormones. Growth factors stimulate cell proliferation and play a pivotal role in increasing growth during development [71,72]. Growth factors are synthesized in most tissues in the body and their action is modulated by a network of molecules that promote cell cycle progression and inhibition of apoptosis [73]. Insulin signalling has been proposed as the mechanism by which a variety of organisms, from beetles to mammals, developmentally regulate expression of exaggerated structures, such as horns and antlers [74]. IGF has also been implicated in numerous cancer phenotypes [73,75,76]. Low levels of IGF-1 may extend the time to malignant proliferation [77]. IGF-1 mutant mouse models live longer and are resistant to cancer [78]. Increased levels of IGF may exacerbate cancer phenotypes through a reduction in apoptosis, an increase in cell turnover or an amplification of effects owing to DNA damage. In humans, mutations in growth hormone receptor genes have been found to be associated with lower cancer risk [79]. Serum from these individuals was found to reduce DNA breaks and increase apoptosis of human epithelial mammary cells in culture [79].

Hormones control many of the physiological processes involved in reproduction from development of sex organs to the timing and frequency of reproduction. Growth factors, and specifically steroid hormones, have been implicated in the growth and progression of many cancers [80–84]. Steroid hormones bind to nuclear receptors within cells and stimulate a complex signalling cascade involved in many cellular actions, including proliferation. Larger body size and larger ornaments or weapons can increase reproductive success; however, this may lead to sexual antagonism and favour reproductive competition over long-term survival [85,86]. One important component of tumour suppression is likely the suppression and slowing of somatic evolution. However, organismal-level selection for growth may relax constraints on somatic evolution within the body. Rapid proliferation may be instantiated through a shorter generation time, which could lead to a faster rate of somatic evolution. A consequence of this may be greater susceptibility to cancer in organisms selected for rapid growth.

### (d) Does reproductive competition influence cancer risk in humans?

Although cancer in the wild is poorly studied, predictions from our model as well as a literature review find examples where sexually selected traits—e.g. body size in fish and antlers in deer—appear to influence cancer susceptibility in these species. Do we see similar trends in humans, a much better studied species with respect to cancer? Our model suggests that differences in overall cancer risk between men and women [87,88] might be partially explained by higher reproductive competition in men than women. Within sexes, reproductive competitiveness appears to be associated with cancer risk for a number of traits. Taller men are reproductively more successful than shorter men, indicating that there is active selection for stature in some human populations [89–91]. In general, taller humans have an increased risk for cancer susceptibility, including melanoma [92] and testicular cancer [93]. Rapid growth prior to reproductive maturity (i.e. becoming bigger faster) is also associated with cancer in humans. Early growth in adolescents and the rate of this growth can influence the risk of prostate cancer in men [94] and breast cancer in women [95]. Outcome of height in an individual depends on many growth factors, including IGF [96]. As noted in §4c, low levels of IGF-1 have been associated with a decreased incidence to several forms of cancer in animals and humans [76,97].

High testosterone levels are associated with increased aggressiveness, high reproductive effort and mating success [85,98–100]. Lifetime exposure to androgens, such as testosterone, appears to be associated with risk of prostate cancer in human males [101]. However, this relationship has been difficult to confirm, as prostate cancer typically has a late age of onset, and testosterone diminishes with age [102]. Additionally, expression levels of androgen receptor, which binds testosterone, may influence fertility in males [85,103] and are associated with an increased risk of prostate cancer [104]. There are open questions whether humans and other species can facultatively trade off between cancer suppression and reproductive competitiveness. Future work on adaptive calibration of physiology based on physical and social environmental inputs during development [105] could provide insights into these open questions.

Within humans, energy budgets can affect the strength of trade-offs between reproduction and somatic maintenance, as seen in figure 3. Forager–horticulturalists, Tsimane, of the Bolivian Amazon live in a resource-limited environment compared with those of industrialized societies. Tsimane men are therefore likely to have stronger trade-offs between reproduction (i.e. testosterone levels) and somatic maintenance (i.e. immune function). Tsimane men, who live in caloric restricted environments with high parasite load, have significantly lower baseline levels of salivary testosterone when compared with age-matched US males [106]. Further, infections, through exposures to pathogens and parasites dramatically decrease testosterone levels [107]. Together, these results suggest that with lower energy budgets there may be a stronger trade-off between somatic maintenance (in the form of immune function) and reproductive competitiveness (as measured by circulating testosterone).

Reproductive competitiveness, including fertility timing, frequency and attractiveness, may influence cancer susceptibility in human females. High oestrogen levels are associated with attractiveness in human females [108–110], and oestrogen is an important component of follicle maturation and oocyte quality [111]. However, the relationship between reproduction and cancer risk in human females is complex and differs for different types of cancer. Even within breast cancer, a recent meta-analysis showed hormone positive breast cancer has been associated with greater exposure to cyclical hormones through earlier menarche, later reproduction and lower parity, whereas hormone negative breast cancer is associated only with early menarche [112]. These findings suggest that hormone positive breast cancer risk may be explained by a mismatch between ancestral reproductive conditions and modern ones, with the result that present-day women experience higher exposure to hormones and suboptimally high rates of cell proliferation, resulting in increased vulnerability to cancer. Hormone negative breast cancer risk, on the other hand, might be mediated by other factors, including perhaps energetic trade-offs favouring reproduction over somatic maintenance.

Other factors that may influence a women's risk for cancer include effects on fertility and pregnancy. As stated earlier, women with BRCA1/2 mutations were shown to have significantly different reproductive profiles than age-matched controls. While BRCA1/2 mutations increase the risk for breast cancer, these women had significantly more children and shorter interbirth intervals [13], suggesting that there may be a fertility advantage for BRCA mutations, possibly accounting for the frequency of BRCA mutations in certain human populations [113]. Additionally, physiological processes involved in gestation, including placentation (as noted in §4a) could influence disease risk. Humans have the most invasive placental type, haemochorial. According to the positive pleiotropy hypothesis, which claims that placental invasiveness should be correlated with susceptibility to metastatic disease [65], a women's cancer risk may be higher with greater depth of placentation. Interestingly, a negative correlation has been found between pre-eclampsia, characterized by abnormally shallow placentation during pregnancy, and breast cancer risk [114,115]. However, this does not hold true for all populations studied [116], and there are likely additional factors influencing cancer susceptibility.

## 5. Conclusion

The role of reproductive investment as a determinant of cancer susceptibility is only beginning to be understood. Here, we contribute to this emerging understanding by presenting a model in which investment in reproductive competitiveness occurs in the presence of a trade-off with investment in cancer defence. We use this model to make predictions about cancer incidence within and across species in various ecological circumstances. Review of relevant literature reveals several examples that are consistent with the model assumptions and predictions. Future comparative oncology and genomic studies will be needed from species with diverse life histories to tease apart the molecular underpinnings that may influence both reproduction and cancer.
